# Supplementary material for: Causal Relationship between Aging and Anorexia Nervosa: A White-Matter-Microstructure-Mediated Mendelian Randomization Analysis
Source: Biomedicines. 2024 Aug 16;12(8):1874. doi: 10.3390/biomedicines12081874 (PMC11351342; doi:10.3390/biomedicines12081874)
Supplement: Supplementary file 1 [file biomedicines-12-01874-s001.zip › Supplementary Figure/Figure S4/Singe SNP plot of MR analysis for TL to ubm-b-1592.pdf]

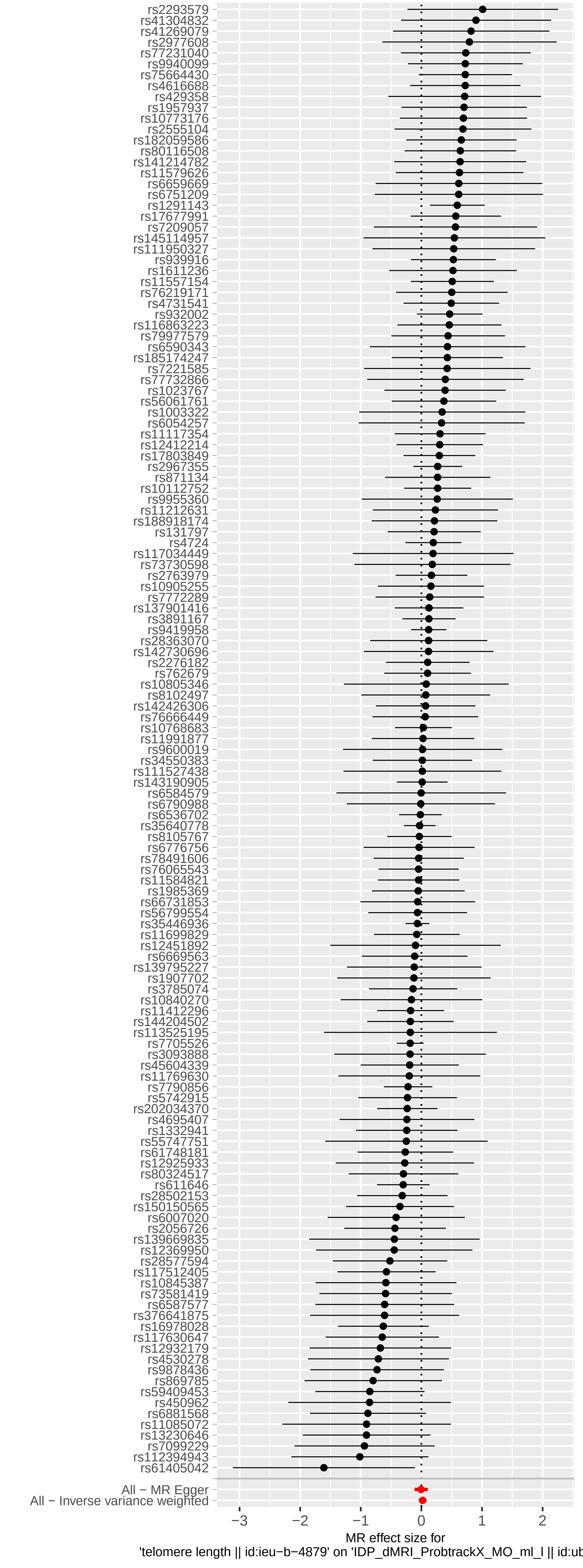
rs2293579
rs41304832
rs41269079
rs2977608
rs77231040
rs9940099
rs75664430
rs4616688
rs429358
rs1957937
rs10773176
rs2555104
rs182059586
rs80116508
rs141214782
rs11579626
rs6659669
rs6751209
rs1291143
rs17677991
rs7209057
rs145114957
rs111950327
rs939916
rs1611236
rs11557154
rs76219171
rs4731541
rs932002
rs116863223
rs79977579
rs6590343
rs185174247
rs7221585
rs77732866
rs1023767
rs56061761
rs1003322
rs6054257
rs11117354
rs12412214
rs17803849
rs2967355
rs871134
rs10112752
rs9955360
rs11212631
rs188918174
rs131797
rs4724
rs117034449
rs73730598
rs2763979
rs10905255
rs7772289
rs137901416
rs3891167
rs9419958
rs28363070
rs142730696
rs2276182
rs762679
rs10805346
rs8102497
rs142426306
rs76666449
rs10768683
rs11991877
rs9600019
rs34550383
rs111527438
rs143190905
rs6584579
rs6790988
rs6536702
rs35640778
rs8105767
rs6776756
rs78491606
rs76065543
rs11584821
rs1985369
rs66731853
rs56799554
rs35446936
rs11699829
rs12451892
rs6669563
rs139795227
rs1907702
rs3785074
rs10840270
rs11412296
rs144204502
rs113525195
rs7705526
rs3093888
rs45604339
rs11769630
rs7790856
rs5742915
rs202034370
rs4695407
rs1332941
rs55747751
rs61748181
rs12925933
rs80324517
rs611646
rs28502153
rs150150565
rs6007020
rs2056726
rs139669835
rs12369950
rs28577594
rs117512405
rs10845387
rs73581419
rs6587577
rs376641875
rs16978028
rs117630647
rs12932179
rs4530278
rs9878436
rs869785
rs59409453
rs450962
rs6881568
rs11085072
rs13230646
rs7099229
rs112394943
rs61405042
All − MR Egger
All − Inverse variance weighted
−3
−2
−1
0
1
2
MR effect size for
'telomere length || id:ieu−b−4879' on 'IDP_dMRI_ProbtrackX_MO_ml_l || id:ub
